# Acute tree nut-triggered food protein-induced enterocolitis syndrome in two infants: a case report

**DOI:** 10.3389/falgy.2026.1926479

**Published:** 2026-08-27

**Authors:** Mariëtte R. Nortier, Anke M. Merks, Anneke A. van Boekholt, Irene C. E. M. Herpertz, Hylke H. A. C. M. van der Heijden, Marjoke M. J. Verweij

**Affiliations:** Department of Paediatrics, VieCuri Medical Centre, Venlo, Netherlands

**Keywords:** atypical FPIES, case report, Children, oral food challenge, tree nuts

## Abstract

**Introduction:**

Food protein-induced enterocolitis syndrome (FPIES) is a non-IgE-mediated food allergy characterised by delayed repetitive vomiting and can be difficult to distinguish from infectious illness or even an IgE-mediated allergy. Tree nuts are uncommon but increasingly recognised triggers, and evidence regarding atypical FPIES with concomitant IgE sensitisation remains limited.

**Case presentation:**

We describe two infants with severe acute reactions to tree nuts who were managed at a paediatric allergy centre. The first, a boy with eczema and cow's milk allergy, developed reproducible delayed vomiting, pallor, and lethargy after cashew ingestion; an oral food challenge (OFC) confirmed atypical FPIES to cashew and required treatment with intravenous fluids, anti-emetics, and corticosteroids. The second, a girl with eczema and a complex allergic phenotype, had IgE-mediated peanut allergy, FPIES to egg and walnut, and presumed atypical FPIES to cashew. Her reactions were characterised by repetitive vomiting, pallor, lethargy, and hypotension with loss of consciousness, requiring intravenous fluid resuscitation. In both cases, interpretation of the symptoms was challenging due to concomitant infections.

**Conclusion:**

These cases add to the limited literature on tree nut-triggered FPIES, illustrate the complexity of atypical FPIES when sensitisation coexists, and highlight challenges in allergen introduction, OFC timing, and management of infants with multiple food allergies.

## 1 Introduction

Food protein-induced enterocolitis syndrome (FPIES) is a non-IgE-mediated food allergy characterised by delayed repetitive vomiting in the absence of cutaneous or respiratory features of IgE-mediated allergy. Treatment with antihistamines and adrenaline, as indicated for IgE-mediated allergic reactions, is ineffective (1).

FPIES mainly affects infants and young children, most of whom outgrow the condition by 5 years of age (2). There is emerging evidence that adults are also affected by this condition, although the presentation and food triggers may differ from those in the paediatric population (3, 4). To date, the pathophysiology is not fully understood.

Since no confirmatory diagnostic tests are available, diagnosis relies on clinical criteria. To meet these criteria, vomiting must occur 1–4 h after ingestion of the suspected food allergen, together with at least three minor criteria: a recurrent episode following ingestion of the same or a different food, lethargy, pallor, hypothermia, hypotension, diarrhoea within 24 h, the need for intravenous fluid support, or emergency medical assessment (1).

A minority of patients with FPIES (9.8%, as mentioned in a recent systematic review) (5) demonstrate IgE sensitisation to the suspected food allergen despite meeting the criteria for FPIES. This is referred to as atypical FPIES (6). It has been suggested that although IgE sensitisation may lead to IgE-mediated allergic reactions, the prevalence of phenotype switching in children to the culprit food is low (13%), implying that most children with FPIES and IgE sensitisation do not develop IgE-mediated symptoms following food exposure during follow-up (5).

Reliable data on the prevalence of FPIES remain sparse. The most commonly reported food allergen to cause an FPIES reaction is cow's milk in the Netherlands and worldwide (7). Other common solid-food triggers may vary by geographic region. Cases with multiple food triggers have also been described, with rates ranging from 47% to 69% in US cohort studies (8, 9). The early introduction of highly allergenic foods, based on the LEAP trial, has increasingly been associated with reports of FPIES triggered by egg, peanut, and other less common foods, such as avocado, banana, and tree nuts, making this an area of ongoing debate (6, 8, 10).

We present two infants referred to our paediatric allergy centre with severe (atypical) FPIES reactions to nuts requiring intravenous fluid resuscitation. The first was a 9-month-old boy with atypical FPIES to cashew. The second was a 6-month-old girl with a complex allergic phenotype, including FPIES to egg and walnut, presumed atypical FPIES to cashew, and IgE-mediated peanut allergy. To our knowledge, FPIES triggered by tree nuts has not been frequently reported to date. These cases highlight the challenges in diagnosis and subsequent management, particularly regarding the early introduction of allergenic foods in infants with multiple food triggers.

## 2 Case presentation

### 2.1 Case 1

A 6-month-old boy with eczema and a family history of atopy was referred for suspected cow's milk allergy. After switching from breast milk to standard formula, he developed vomiting, erythema, and discomfort. A double-blind, placebo-controlled oral food challenge (OFC) confirmed cow's milk allergy. Cow's milk was subsequently eliminated from his diet and replaced with an extensively hydrolysed formula.

Parents were advised to introduce peanuts and eggs at home, following Dutch early-introduction guidelines, and were also encouraged to introduce tree nuts at home under the guidance of a paediatric allergy dietitian. At 9 months of age, following the successful home introduction of peanut, egg, and almond, his parents contacted the allergy centre after he appeared to react to cashew on three occasions. Each episode was characterised by repetitive vomiting. The first two episodes were mild, occurred after ingestion of two teaspoons of cashew butter, and coincided with symptoms of a viral infection. During the third exposure, when he was otherwise well, he received one teaspoon in the morning and two teaspoons in the afternoon. He developed repetitive vomiting 2–3 h later, accompanied by pallor and lethargy but without diarrhoea, and recovered fully by the next morning.

FPIES to cashew was suspected, with no other features suggestive of an IgE-mediated allergy. Skin prick testing showed sensitisation to cashew (with a 3.5 mm reaction), pistachio (2.5 mm), walnut (3.5 mm), and cow's milk (7 mm), while testing for hazelnut and soya was negative. Hazelnut was successfully introduced at home at 11 months of age; walnut and cow's milk were (re)introduced following negative OFCs at age 13 and 14 months of age, respectively.

At 16 months of age, a 2-day open oral food challenge to cashew was performed using an IgE-mediated food allergy dosing protocol in accordance with PRACTALL (11), given the presence of cashew-specific IgE sensitisation, with a dose interval of 1 h and an observation period of 4 h following the final dose on each day. In cases of suspected FPIES with IgE sensitisation, our local practice is to extend both the dosing interval and the observation period. On day 1, he started vomiting 30 min after a cumulative dose of 443 mg of cashew protein. Repeated doses of oral ondansetron were given without an adequate clinical response. He appeared pale and lethargic, with no symptoms suggestive of an IgE-mediated allergy, such as visible skin/mucosal reactions or respiratory symptoms. However, given the rapid onset of symptoms and his sensitisation to cashew, an IgE-mediated reaction was suspected and therefore treated with intramuscular adrenaline and an antihistamine. Despite these interventions, vomiting persisted. He eventually recovered after treatment with intravenous dexamethasone and fluids. His vital signs, including temperature, remained stable throughout. The neutrophil count rose from 2.0 × 10^9 ^/L to 13.1 × 10^9 ^/L 3.5 h after the onset of the reaction, while tryptase levels remained low. He was discharged the following day in good clinical condition and did not develop diarrhoea.

He met the diagnostic criteria for atypical FPIES (Table 1) and was provided with a personalised emergency plan. The major criterion was met by assessing the reaction time following the initial doses.

**Table 1:** Case 1.

Age (months)	Event	Amount of food (g) Cumulative protein dose (mg)	Time of reaction from the first dose Time of reaction from the final dose	Symptoms	Treatment	Change in neutrophil count (x10^9^/L) Start—3.5 h after reaction onset	Sensitisation Skin prick test and/or sIgE	FPIES criteria	Sampson score
9	Possible home reaction to cashew	3 teaspoons (10 g) 2,000	2–3 h from the final dose	Vomiting Pallor Lethargy	–	–	Cashew 3.5 mm Pistachio 2.5 mm	Major: vomiting 1–4 h after ingestion Minor: pallor, lethargy	
13	OFC walnut	22.09 3,443	–	–	–	–	3.5 mm	–	–
14	OFC cow's milk	233 mL 6,332	–	–	–	–	7 mm	–	–
16	OFC cashew in the hospital	2.07 443	First: 4.5 h Final: 30 min	Persistent vomiting Pallor Lethargy	Ondansetron orally x3 Levocetirizine orally Adrenaline IM Dexamethasone orally Dexamethasone IV Fluids IV	2.0–13.1	Cashew 6.4 kU/L	Lethargy Pallor Increased neutrophil count	

He is now nearly 3 years old and doing well, having eliminated both cashew and pistachio from his diet because of cross-reactivity. A repeat challenge is planned to assess whether tolerance has been acquired.

### 2.2 Case 2

A 5-month-old girl with eczema and a family history of atopy was referred following a reaction during the home introduction of peanut, performed in accordance with Dutch early-introduction guidelines. She was a thriving infant and receiving a standard antireflux formula because of frequent posseting. Several fruits and vegetables had already been introduced successfully.

**Figure 1:**
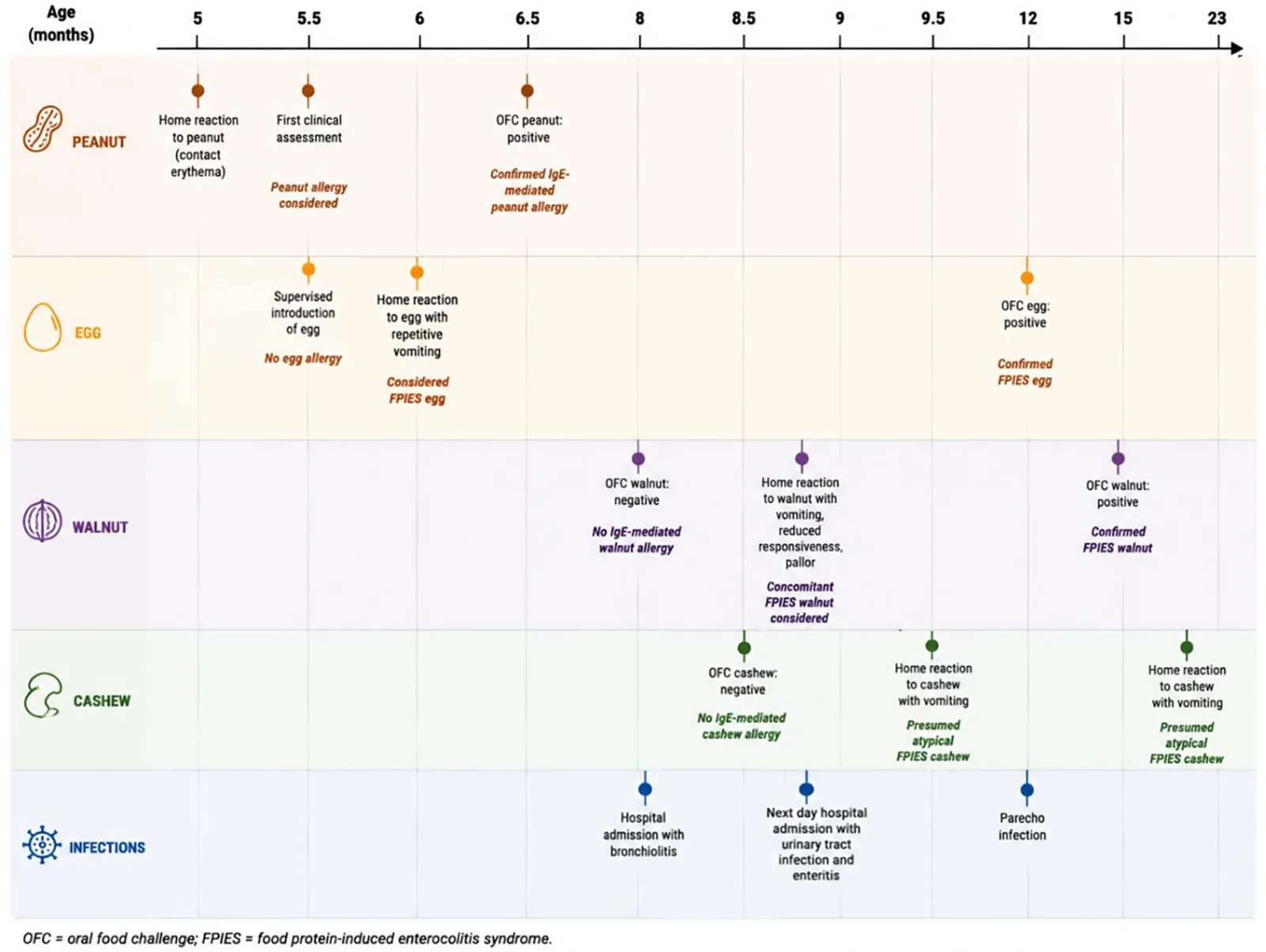
Timeline of case 2. OFC, oral food challenge; FPIES, food protein-induced enterocolitis syndrome.

For clarity, this case is presented according to the individual allergens. An overview of the chronological course of events is presented in Figure 1, while the results of the diagnostic evaluation and OFC are summarised in Tables 2, 3, respectively.

**Table 2:** Case 2 investigations.

Age (months)	Investigation	Result
5.5	Skin prick test	Peanut 5.5 mm Egg, walnut, almond, hazelnut, cashew 0 mm
9	Urine	*E. coli* positive
9	Feces PCR	Positive for rotavirus, adenovirus, and norovirus
9	Blood—specific IgE (<0.35)	Peanut: 6.2 kU/L Pistachio: 1.1 kU/L Brazil nut: 0.95 kU/L Cashew: 0.80 kU/L Hazelnut: 0.45 kU/L Egg 0.19 kU/L Almond: 0.16 kU/L Macadamia: 0.04 kU/L Walnut: 0.01 kU/L Pecan: <0.01 kU/L
12	Blood before OFC egg Specific IgE (<0.35)	Neutrophil count: 2.6 × 10^9 ^/L (1.5–8.0) Egg: 0.27 kU/L
	Blood after OFC egg	Neutrophil count: 6.4 × 10^9 ^/L (1.5–8.0)
12	Faeces PCR	Positive for parechovirus
15	Blood—before OFC walnut specific IgE (<0.35)	Neutrophils: 3.3 × 10^9 ^/L (1.5–8.0) Peanut: 5.0 kU/L Egg: 1.1 kU/L Pistachio: 0.42 kU/L Cashew: 0.29 kU/L Hazelnut: 0.28 kU/L Brazil nut: 0.28 kU/L Macadamia: 0.04 kU/L Almond: 0.04 kU/L Walnut: 0.02 kU/L Pecan: 0.01 kU/L
	Blood—after OFC walnut	Neutrophils: 14.7 × 10^9 ^/L (1.5–8.0)

OFC, oral food challenge; PCR, polymerase chain reaction.

Reference values are in parentheses.

**Table 3:** Oral food challenges for case 2.

Age (months)	Event	Amount of food (g) Cumulative protein dose (mg)	Time of reaction from the first dose Time of reaction from the final dose	Symptoms	Treatment	Change in neutrophil count (x10^9^/L) Start—4 h after the reaction onset	Sensitisation Skin prick test and/or sIgE	FPIES criteria	Sampson score
5.5	Supervised egg introduction	25.5 3,185	–	–	–	–	–	–	–
6.5	OFC peanut	17.77 4,443	First: 6 h Final: 2 h	Sneezing Blocked nose Vomiting once Erythema Swollen ears	Antihistamine	–	SPT 5.5 mm	–	2
8	OFC walnut	22.09 3,443	>12 h after final dose	Vomiting once	–	–	SPT 0 mm	–	–
8.5	OFC cashew	15.77 3,443	–	–	–	–	SPT 0 mm	–	–
12	OFC egg	35.65 4,443	First: 4 h Final: 30 min	Vomiting Pallor Lethargy Hypotension Diarrhoea	Granisetron IV 2x Intravenous fluids Dexamethasone IV	2.6–6.4	sIgE 0.27 kU/L	1, 2, 3, 4, 6	–
15 m	OFC walnut	9.09 1,443	First: 3 h 15 min Final: 25 min	Vomiting Pallor Lethargy and loss of consciousness Hypotension	Granisetron IV 2x Intravenous fluids Dexamethasone IV	3.3–14.7	sIgE 0.02 kU/L	1, 2, 4, 6	–

OFC, oral food challenge; SPT, skin prick test; sIgE, specific IgE.

1, lethargy; 2, pallor; 3, diarrhoea; 4, hypotension; 5, hypothermia; 6, increased neutrophil count ≥ 1.5 × 10^9 ^/L above the baseline count.

#### 2.2.1 Peanut

Peanut caused immediate perioral and contact erythema during home introduction, without gastrointestinal symptoms. At clinic assessment, skin prick testing showed a 5.5-mm wheal to peanut, while tests to egg, walnut, hazelnut, almond, and cashew were negative.

An OFC to peanut, performed nearly 6 weeks after the initial reaction at home, was positive 2 h after ingestion of a cumulative dose of 4,443 mg of peanut protein, with a grade 2 Sampson Score reaction, characterised by persistent sneezing, a blocked nose, a single episode of large-volume vomiting, erythema of areas of skin without direct food contact, and swelling of the ears. She was treated with an antihistamine and diagnosed with IgE-mediated peanut allergy. Peanut-specific IgE levels were 6.2 kU/L at 3 months and 5.0 kU/L at 9 months after the OFC.

#### 2.2.2 Egg

Home introduction of egg was first attempted 1 week after the initial peanut reaction. She tolerated one teaspoon of cooked egg on day 1, but developed repetitive vomiting after consuming the same amount on day 2, without appearing unwell or showing signs of infection. As she was known to vomit easily, her parents were not immediately concerned.

Following negative skin prick testing, a supervised introduction at the clinic was tolerated up to 3,185 mg of egg protein, and home introduction was therefore continued. This initially went well. However, 2 weeks later, she developed repetitive vomiting 75 min after eating half an egg, accompanied by pallor and restlessness but no other symptoms. FPIES to egg was suspected, and egg was subsequently avoided pending clinic review.

The parents later questioned whether the reactions at home were truly consistent with FPIES, given her tendency to vomit easily. They were concerned that prolonged avoidance might increase the risk of IgE-mediated egg allergy; therefore, a formal challenge was performed 6 months later, at 12 months of age. Because she had experienced multiple severe reactions to different foods in the interim, a more cautious 2-day OFC was planned, with intravenous access in place. Day 1 followed the standard PRACTALL protocol, and day 2 was intended to consist of a single age-appropriate serving of one whole egg.

On the day of the challenge, she had a mild cold, with only a slightly runny nose, but was otherwise well and was considered fit enough to proceed. On day 1, 30 min after receiving a cumulative dose of 4,443 mg of egg protein, she developed repeated vomiting that persisted despite intravenous granisetron. At 90 min, she became pale and lethargic and experienced a brief hypotensive episode, without hypothermia. Intravenous fluids and a repeat dose of granisetron were administered. Recovery followed after intravenous dexamethasone.

Blood samples obtained 4 h after symptom onset showed a rise in neutrophil count from 2.6 to 6.4 × 10^9 ^/L, while tryptase levels remained low. Later that day, she developed diarrhoea, which lasted for several days; stool testing was positive for parechovirus. Egg-specific IgE, measured when intravenous access was established before the challenge, was 0.27 kU/L (negative <0.35 kU/L), compared with 0.19 kU/L 3 months earlier; this then rose to 1.1 kU/L 3 months after the challenge. The major criterion was met by assessing the reaction time following the initial doses. She was diagnosed with acute FPIES to egg, although interpretation of the challenge was limited by the possibility of a concurrent infection.

#### 2.2.3 Walnut

Walnut and cashew had been eaten frequently at home by the parents. However, because of parental anxiety, supervised oral food challenges to both walnut and cashew were arranged.

At 8 months of age, a walnut OFC was considered negative after ingestion of 3,443 mg of walnut protein. However, 12 h after the final dose, she vomited during the night, without other features suggestive of either an IgE-mediated allergic reaction or FPIES. Over the following days, she developed a high fever, a viral exanthema, dyspnoea, persistent vomiting, and diarrhoea, and was ultimately admitted to the paediatric ward with bronchiolitis.

Two weeks after discharge, walnut was reintroduced at home. Two hours after the penultimate dose, she developed repetitive vomiting, reduced responsiveness with loss of muscle tone, and marked ash-grey colour change. By the time the ambulance arrived, she had recovered. She was treated by the ambulance crew with antihistamines and antiemetics. There were no features suggestive of an IgE-mediated reaction. She was admitted for observation and discharged the following day.

She was readmitted the next day with recurrent vomiting, diarrhoea, cough, and malodorous urine. Investigations showed an *Escherichia coli* urinary tract infection and enteritis with adenovirus, norovirus, and rotavirus detected. Although this cluster of symptoms over the preceding weeks was thought to be explained by multiple intercurrent infections rather than walnut exposure alone, her parents were advised to avoid walnut, and a repeat OFC was scheduled for 15 months of age.

During the repeat challenge, performed according to the PRACTALL protocol, she developed repetitive vomiting 25 min after ingesting 1,443 mg of walnut protein. She then became hypotensive and tachycardic, with several episodes of loss of consciousness, requiring repeated fluid boluses, intravenous granisetron, and dexamethasone. Her neutrophil count rose from 3.3 × 10^9^ /L at baseline to 14.7 × 10^9^ /L 4 h after symptom onset, while tryptase levels remained low. There was no evidence of sensitisation: walnut-specific IgE was 0.02 kU/L on the day of the second challenge, compared with 0.01 kU/L 1 month after the first challenge. A diagnosis of FPIES to walnut was made.

#### 2.2.4 Cashew

Ten days after her admission with bronchiolitis, and 2 weeks after the initial negative walnut OFC, a cashew OFC was negative after she tolerated a cumulative dose of 3,443 mg of cashew protein.

Given her recent illness with multiple infections, her parents delayed the home introduction of cashew for 1 month. She ate a total of seven cashews mixed with fruit and developed repetitive vomiting 3 h later, without cutaneous or respiratory symptoms. Ondansetron and antihistamines administered at home were ineffective. She remained alert and otherwise well, with no pallor or no signs of infection. She vomited again in the emergency department, where she received oral granisetron and was admitted for observation.

Blood tests during this admission showed sensitisation to cashew and pistachio, with specific IgE levels of 0.80 and 1.1 kU/L, respectively. A presumed diagnosis of atypical FPIES to cashew was made. Six months later, cashew- and pistachio-specific IgE levels had decreased to 0.29 and 0.42 kU/L, respectively.

A year later, when she was nearly 2 years old, accidental exposure after licking a vegan ice cream containing cashew resulted in two episodes of vomiting 2 h later, which responded to ondansetron. She did not require hospital attendance.

#### 2.2.5 Patient experience

Following the severe reaction during the second walnut OFC, a shared decision was made with her parents not to proceed with further oral food challenges for the time being. Over the preceding 9 months, she had experienced significant reactions during three hospital-based challenges and on four occasions during home introduction, some of which occurred in the context of intercurrent infections. This had been highly distressing for the family, and her mother required Eye Movement Desensitisation and Reprocessing (EMDR) therapy. The parents wanted their daughter to live as normally as possible without becoming overprotective, but at the same time felt a strong need to remain constantly vigilant to keep her safe.

They understandably remain fearful of introducing new foods. Her diet currently excludes nuts, peanut, and egg, although trace exposure is not avoided. The introduction of other high-risk allergens, including soya, has been paused, although she tolerates small amounts of processed soya. Her diet currently includes dairy, fish, meat, pulses, seeds including sesame, pine nuts, a wide variety of fruits and vegetables, potatoes, and grains including wheat, quinoa, and rice, as well as a variety of spices and fresh herbs. The expertise of a paediatric allergy dietitian is pivotal in maintaining optimal nutrition and promoting dietary diversity to ensure adequate growth in (young) children. In case 2, particular attention was given to ensuring sufficient intake of unsaturated fats, given the exclusion of peanuts and tree nuts from her diet.

## 3 Discussion

Diagnosing FPIES remains challenging because its symptoms are often non-specific and frequently misdiagnosed. Appropriate management depends on accurate recognition, as treatment primarily consists of supportive care, including antiemetics and fluid resuscitation. Management is further complicated in atypical FPIES, where the presence of specific IgE to the trigger food may result in IgE-mediated symptoms requiring adrenaline and/or antihistamine. These overlapping presentations make it difficult for caregivers to distinguish between reaction types and choose the right treatment, highlighting the need for clear and practical emergency guidance.

### 3.1 Tree nuts as emerging FPIES triggers

These two cases illustrate several important aspects of the diagnosis and management of FPIES. They also add to the limited but growing literature describing tree nuts as potential FPIES triggers.

FPIES triggers vary geographically and appear to reflect regional dietary practices. Cow's milk is a major trigger worldwide, rice is commonly reported in the United States and Australia, and fish is a frequent trigger in Europe. Reports of FPIES to egg and peanut have increased in recent years, coinciding with recommendations for early introduction of allergenic foods (6, 12). Although nut-triggered FPIES remains uncommon, it may be increasingly recognised. In an Australian retrospective study, Baldwin et al. reported 10 cases of nut-related FPIES, of which 9 involved peanut and 1 involved a tree nut (hazelnut) (10). In a large paediatric cohort from the United States, only 3% of cases were attributed to nuts (8). Published case reports of tree nut FPIES remain rare. A reviewing of the literature identified one case report of walnut-triggered FPIES, with a different phenotype and a later onset at 3 years of age (13).

Both patients had atypical FPIES to cashew. This was a presumed diagnosis for case 2, who also had FPIES to walnut and egg. As recommendations increasingly support the early introduction of tree nuts alongside peanut and egg, an important question is whether this timing overlaps with a period of heightened susceptibility to FPIES during infancy (14)? Although a case series of peanut-induced FPIES suggested such an association, a large prospective cohort study of infants at high risk of IgE-mediated peanut allergy reported a prevalence of peanut-induced FPIES of 0.3%, consistent with population-based estimates of paediatric FPIES (15, 16). More studies are needed to determine whether early introduction leads to an increased risk of developing FPIES.

### 3.2 Possible role of intercurrent infections

In both cases, some reactions occurred in temporal proximity to intercurrent infection, whereas other reproducible reactions occurred when the children were clinically well. The concomitant infections complicated the interpretation of symptoms, which could have stemmed from the infection alone or from a combination of the infection and an adverse food reaction.

It has been proposed that viral infections may act as cofactors or immune-modulating events that unmask FPIES. A recent case report on FPIES following COVID-19 infection described such an association in a susceptible infant (17), although the evidence remains limited. Current hypotheses include impaired epithelial tolerance leading to innate immune hyperactivation, with possible contributions from microbiome dysbiosis and genetic susceptibility, resulting in impaired epithelial integrity and irregular innate immune priming (18, 19).

These cases cannot determine whether infection acted as a cofactor in the development of FPIES or as its trigger.

### 3.3 Implications for food introduction and timing of oral food challenges

These cases also raise questions for clinical care. What advice should we give caregivers about the timing of the introduction of allergenic foods in relation to infections or recent adverse food reactions? OFCs should be deferred in the presence of acute infection. However, many parents report that their children have a persistently runny nose despite otherwise being well, making it difficult to judge whether an active viral infection is developing. How long should a child be well before introduction is attempted? During the first year of life, when many new foods are introduced, frequent infections are common. As a result, some caregivers may really struggle to find a suitable window not only for introducing foods but also for maintaining regular exposure once introduced.

Much also remains unknown about the natural history of FPIES, particularly for uncommon triggers such as tree nuts. The timing of OFC depends on the clinical aim: to confirm the diagnosis when uncertainty remains, or to assess whether the child has developed tolerance. In both of our cases, some challenges were performed approximately 6 months after the most recent reaction. Whether a longer interval would have been preferable—for example, 1–1.5 years, as suggested by Nowak et al. (1)—remains unclear. However, there was either doubt about the diagnoses of FPIES or a need for early introduction because of risk factors for the development of IgE-mediated food allergy. Decisions about whether and when to challenge were made by balancing competing uncertainties; data to support decision-making remains very limited.

One potentially reassuring finding, as reported in a recent systematic review and meta-analysis by Phelan et al. (5), is that the overall phenotype switch from acute FPIES to IgE-mediated food allergy, even in those sensitised, is uncommon (13%). A comparable rate of 9.2% was reported in a retrospective study assessing the significance of egg sensitisation in paediatric egg FPIES (20).

### 3.4 Strengths

For both patients, diagnoses were supported by comprehensive allergy assessment, pathogen screening, and multiple standardised oral food challenges.

### 3.5 Limitations

The contribution of intercurrent infection and early allergenic food introduction to the development of FPIES cannot be established from these cases.

## 4 Conclusion

Tree nuts are uncommon but recognised triggers of acute FPIES in infancy, including atypical presentations with coexisting IgE sensitisation. These cases highlight the ongoing diagnostic and management challenges of FPIES, particularly when there is concurrent concern about IgE-mediated allergy. They also highlight important gaps in the current evidence regarding the role of infections and early introduction of allergenic foods in the development of FPIES, the optimal timing of oral food challenges, and the natural history of tree nut-triggered FPIES. Further research is needed to address these uncertainties and to better support caregivers in making informed decisions about their child's care.

## Data Availability

The original contributions presented in the study are included in the article/Supplementary Material, further inquiries can be directed to the corresponding author.
